# Hematological and Biochemical Changes in Dogs Naturally Infected With *Dirofilaria repens*

**DOI:** 10.3389/fvets.2020.00590

**Published:** 2020-09-10

**Authors:** Magdalena E. Wysmołek, Artur Dobrzyński, Ewa Długosz, Michał Czopowicz, Marcin Wiśniewski, Piotr Jurka, Maciej Klockiewicz

**Affiliations:** ^1^Division of Parasitology, Department of Preclinical Sciences, Institute of Veterinary Medicine, Warsaw University of Life Sciences-SGGW, Warsaw, Poland; ^2^Department of Small Animal Diseases and Clinic, Institute of Veterinary Medicine, Warsaw University of Life Sciences-SGGW, Warsaw, Poland; ^3^Division of Veterinary Epidemiology and Economics, Institute of Veterinary Medicine, Warsaw University of Life Sciences-SGGW, Warsaw, Poland

**Keywords:** *Dirofilaria repens*, subcutaneous dirofilariosis, dog, hematology, biochemistry

## Abstract

Subcutaneous dirofilariosis is a zoonotic disease emerging throughout Europe caused by the filarial nematode *Dirofilaria repens*. Despite its increasing prevalence, there is a large gap in knowledge of the impact of the parasite on the host. Currently classified as being non-pathogenic, recent evidence suggests that skin dirofilariosis is associated with dermatological conditions including concomitant pruritus, neoplastic processes, inflammation, and even blindness in dogs and humans. The aim of this study was to determine if natural canine *D. repens* infection leads to biological changes in the canine host. In a real-life veterinary clinic setting, animals are often presented to clinicians for unrelated issues, and *D. repens* is incidentally identified during inspection. As such, we compared hematological and biochemical parameters of 218 uninfected and 197 dogs naturally infected with *D. repens*. Interestingly, animals infected with *D. repens* had lower numbers of lymphocytes (*p* < 0.001), red blood cells (*p* < 0.001), and thrombocytes (*p* = 0.025), decreased hematocrit (*p* < 0.001), and increased alkaline phosphatase (*p* = 0.016) and creatinine activity (*p* = 0.023) compared to uninfected dogs. We further selected a subpopulation of 214 dogs having *prima facie* hematological and biochemical results within normal reference ranges to evaluate the effect of *D. repens* infections in seemingly healthy dogs. Among these patients, 93 dogs infected with *D. repens* had lower numbers of lymphocytes (*p* = 0.031), red blood cells (*p* = 0.025), and hematocrit (*p* = 0.002), higher glucose levels (*p* = 0.023), and border line elevated alkaline phosphatase levels (*p* = 0.054) compared to 121 uninfected animals. Despite being categorized as asymptomatic, we have observed hematological and biochemical changes associated with *D. repens* infections in dogs, and our data suggest that dirofilariosis may induce a state of chronic stress. These results link the presence of skin dirofilariosis to biological changes in the canine host, suggesting a mechanism for pathogenicity and shedding new light on the host–parasite relationship.

## Introduction

*Dirofilaria immitis* and *Dirofilaria repens* are both filarial nematodes that have zoonotic potential and cause canine heartworm disease and skin dirofilariosis in dogs, respectively. Despite its predominance throughout Europe and being the primary causative agent of human dirofilariasis, *D. repens* has received much less attention and study than *D. immitis* (1).

Briefly, dirofilariosis is a vector-borne disease transmitted by mosquitos. Mosquitos uptake microfilariae (Mf) circulating in the bloodstream of a definitive host during blood meals. After 2 weeks, Mf develop into infective larvae and are injected into a new host when the mosquito takes another blood meal. In the new host, the L3 larvae penetrate into subcutaneous tissue where they mature into adult skin filarial worms, copulate, and release Mf into the circulating bloodstream. Adult *D. repens* can survive and reproduce in a host for as long as 5–10 years (2) and may actively migrate within the host tissues during this period what considerably hampers their detection (3).

*Dirofilaria* spp. infections can be divided into microfilaremic and amicrofilaremic (occult). Microfilaremic infections are diagnosed using a modified Knott's test or by examining blood smears; Mf can be differentiated between species by morphology or using multiplex PCR. Although *D. immitis* amicrofilaremic infection may be diagnosed using a standard commercial test for adult female parasite antigens, there is no such rapid test available for the detection of *D. repens* occult infection in dogs (1).

In humans, the adult worms can migrate to subcutaneous tissue of different parts of the body (commonly in the head, mainly the periorbital region and neck), but they can also localize in the epididymis, the spermatic cords, the lungs, the breasts, the visceral cavity, or under the conjunctive tissue, lymph nodes, and muscles (4). In dogs, they are usually incidentally found in the scrotum during castration, but they can reach other locations including the periorbital or ectopic regions, such as mesenteric tissue and pelvic cavity (1). Infection with *D. repens* is usually considered asymptomatic, but a growing body of evidence suggest that it can cause significant morbidity, and increasing cases have been reported of severe disease leading to liver or kidney failure (5). *Dirofilaria repens* infections were also connected to neoplastic, sometimes malignant, processes in dogs (6, 7) and humans (8). Furthermore, the presence of adult *D. repens* and circulating Mf in blood or parenchymal organs can influence the course of coexisting diseases in infected animals (5, 9). General emaciation has been observed during massive *D. repens* infections associated with peritonitis, jaundice, degeneration of the liver, and renal failure (5, 10). In such conditions, numerous Mf were found in histopathology of internal organs (5–7). Microfilariae remaining in capillaries or disseminated into parenchymal tissues may be involved in the pathogenesis of tissue lesions and progress of the ongoing disease (5).

Infections presenting with conspicuous clinical signs and a severe course of disease progression are clear indicators to employ anthelmintic intervention for the welfare of infected host, but those that are asymptomatic should not be neglected. Untreated infection increases the zoonotic risk (1), can lead to the development of predisposition to other diseases, and may cause silent undiagnosed morbidity. Unfortunately, there are currently no biochemical markers to assess the impact of *D. repens* infections.

The aim of this study was to determine if natural *D. repens* infection leads to biological changes, which may be detected in a real-life clinical situation, where animals are presented to clinicians for unrelated issues, and *D. repens* comorbidity is incidentally identified during inspection.

## Materials and Methods

### Study Design

**Figure 1:**
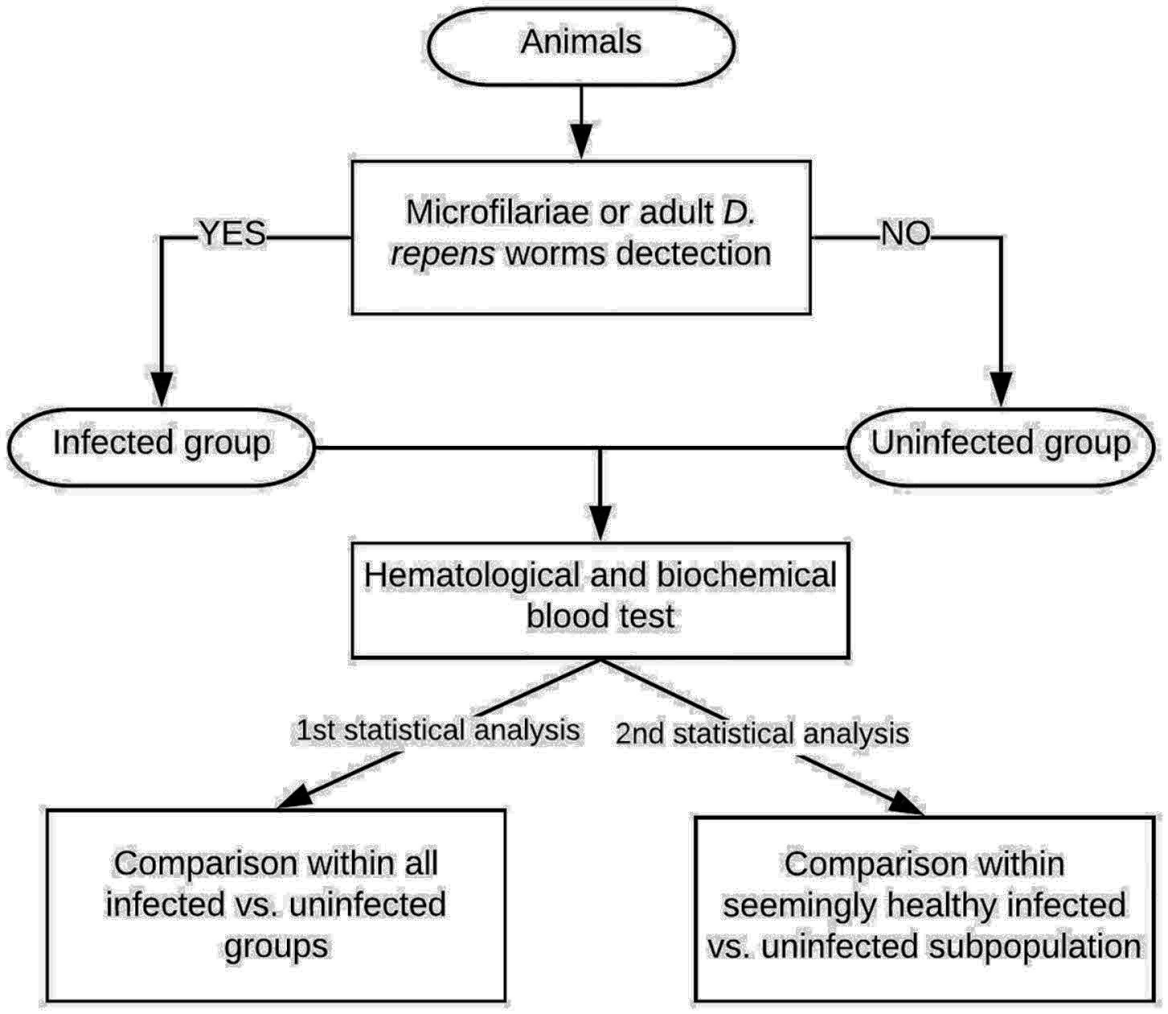
Study design: The examined dogs were classified into the infected group based on the presence of Mf in blood smear/positive result of multiplex PCR/adult *D. repens* parasites found during surgery. Then, blood tests were performed on all individuals. The first statistical analysis included all patients, while the second analysis was performed on a seemingly clinically healthy subpopulation of dogs having blood check-up results within normal reference ranges.

The flow chart of the study design is shown in Figure 1.

#### Animals

A total of 415 dogs of different breeds, sexes, body weights, health, and breeding status, aged 1–17 years were admitted to the study. Those patients were presented to veterinary clinics for regular health issues and/or because they used to live with dogs previously infected with *D. repens*. All examined dogs lived in Poland and had never left the country before the study.

#### Sampling

Patients underwent routine physical examination and blood test. *Dirofilaria repens* infection was diagnosed by direct analysis of blood smears, stained blood smears, adult worm examination (if obtained surgically, *n* = 24), and multiplex PCR.

Five dogs with confirmed *Babesia canis* infections were excluded from the data analysis to obtain the pattern characteristic for only one vector-borne disease.

Dogs were assigned to the infected group if at least one of the three parasitological examinations was positive for *D. repens*, and the rest of animals with negative results were qualified to the uninfected group.

We initially compared the parameters within all 415 dogs; 197 were infected, and 218 were uninfected with *D. repens*. Then, within the entire study population, we selected a subpopulation of 214 (98 infected and 112 uninfected) dogs having hematological and biochemical blood parameters within normal reference ranges. We believe that this group represents the asymptomatic patients diagnosed with *D. repens* incidentally.

### Laboratory Analysis

#### Parasitological Examination

Adult worms isolated during surgical procedures were examined under a light microscope and, based on their morphology, were identified as *D. repens*. Microfilariae were detected by microscopic examination of blood smear. Subsequently, genomic DNA was isolated from blood samples using the Blood Mini kit (AA Biotechnology, Poland) and used as a template for multiplex PCR in order to discriminate between *D. immitis* and *D. repens* species according to Gioia et al. (11). PCR results were analyzed by electrophoresis on 2% agarose gels. All infected dogs tested positive for *D. repens* and none for *D. immitis*.

#### Hematological and Biochemical Analysis

Hematological and biochemical analysis were performed in commercial veterinary diagnostic laboratories. The following standard blood check-up parameters were determined and evaluated according to reference ranges (Table 1): white blood cell count (WBC), neutrophil count, lymphocyte count, eosinophil count, red blood cell count (RBC), packed cell volume (hematocrit, HCT), mean corpuscular volume (MCV), platelet count (PLT), enzyme activity [alanine aminotransferase (ALT), aspartate aminotransferase (AST), alkaline phosphatase (ALP)], and metabolite concentration [total protein (TP), glucose, urea, and creatinine].

**Table 1:** Normal reference values of investigated hematological and biochemical parameters.

	**Unit**	**Reference intervals**
RBC	T/L	5–8
HCT	%	35–60
MCV	fL	55–80
PLT	G/L	100–500
WBC	G/L	5–16
AST	U/L	<100
ALT	U/L	<100
ALP	U/L	<200
TP	g/L	50–80
Glucose	mmol/L	3.8–6.7
Urea	mmol/L	3–12
Creatinine	μmol/L	<150

### Statistical Analysis

The number of dogs included in both statistical analyses with respect to their hematological and biochemical blood parameters differed due to missing data of some individuals.

Data were presented as a mean ± standard deviation (SD) or a median and interquartile range (IQR) and compared between groups using the unpaired-sample Student's *t*-test or Mann–Whitney U-test depending on the asymmetry of a variable distribution assessed on the basis of histograms. The range was always presented. Categorical variables were presented as the number and proportion of dogs in a given group and compared between groups using the Pearson's chi-square test. Ninety-five percent confidence intervals (95% CI) for proportions were calculated with the Wilson score method (12). A two-tailed significance level (α) was set at 0.05. Total study population analysis was performed in Statistica 12 (StatSoft Inc., Tulsa, OK). The analysis of the subpopulation of dog parameters within normal reference range was performed in TIBCO Statistica 13.3.0 (TIBCO StatSoft Inc., Palo Alto, CA).

#### The Analysis of Check-Up Parameters Within the Total Study Population

This included 415 dogs: 225 males (54.2%) and 190 females (35.8%). Their age ranged from 8 months to 17 years with the median of 7 years (IQR from 4 to 10 years) and did not differ between sexes (*p* = 0.655). There were 56 castrated dogs (13.5%) and 112 (27.0%) pedigree dogs. Their body weight varied from 1 to 75 kg with the median of 16 kg (IQR from 10 to 25 kg).

Two hundred eighteen dogs (52.5%) were allocated to the infected group and 197 (47.5%) to the uninfected group, respectively. Infected dogs were significantly heavier (*p* = 0.041) and more often belonged to a particular breed (*p* < 0.001).

#### The Analysis of Parameters Within the Subpopulation Having Results Within Normal Range Values

This included 214 out of 415 dogs with blood check-up within or only slightly (by not more than 25% of the upper limit) deviated from the reference intervals. There were 121 males (56.5%) and 93 females (43.5%). Their age ranged from 1 to 14 years with the arithmetic mean (SD) of 6.8 (3.2) years and did not differ significantly between males and females (*p* = 0.190). There were 171 cross-breed dogs (79.9%) and 43 pedigree dogs (20.1%) belonging to the following breeds: German shepherd (*n* = 9), Yorkshire terrier (*n* = 5), Border collie (*n* = 4), Labrador retriever (*n* = 3), French bulldog (*n* = 3), Chesapeake retriever (*n* = 2), Boxer (*n* = 2), Bavarian mountain dog (*n* = 2), Belgian shepherd (*n* = 2), and Alaskan malamute, Amstaff, Polish hound, Black Russia terrier, Akita Inu, Dachshund, Chinese Crested dog, Bernese Mountain dog, Pug, Poodle, and Welsh terrier (*n* = 1 each).

## Results

We first compared blood hematology and biochemistry results within the entire study population. Dogs infected with *D. repens* compared to uninfected individuals had significantly lower lymphocyte count (*p* < 0.001), RBC (*p* < 0.001), Ht (*p* = 0.001), and thrombocyte count (*p* = 0.025) and higher ALP (*p* = 0.016) and creatinine (*p* = 0.023) activity (Table 2).

**Table 2:** Hematological and biochemical parameters in the total study population.

**Parameter**	**Infected**		**Uninfected**		
**Hematology**	***n***	**Mean ± SD (range)**	***n***	**Mean ± SD (range)**	***t*-test *p*-value**
WBC (G/L)	206	14.9 ± 9.2 (4.7–84.7)	192	14.5 ± 6.2 (3.5–50.4)	0.640
Neutrophils (G/L)	177	10.4 ± 6.5 (1.6–63.8)	189	10.1 ± 5.4 (1.4–39.8)	0.636
Lymphocytes (G/L)	177	2.4 ± 1.5 (0–9.0)	189	3.1 ± 1.6 (0–9.4)	<0.001
Eosinophils (G/L)	177	0.9 ± 0.9 (0–5.1)	189	0.9 ± 1.0 (0–6.2)	0.904
Erythrocytes (T/L)	206	6.7 ± 1.1 (3.6–10.0)	191	7.1 ± 1.0 (3.7–9.9)	<0.001
Haematocrit (%)	206	43.2 ± 6.8 (22–60)	192	45.5 ± 6.3 (22–65)	0.001
MCV (fl)	206	64.8 ± 4.5 (36–77)	192	64.5 ± 3.5 (53–79)	0.404
PLT (G/L)	206	316 ± 160 (13–1000)	192	351 ± 169 (33–1000)	0.025
**Biochemistry**	***n***	**Median, IQR (range)**	***n***	**Median, IQR (range)**	**Mann-Whitey** ***U*****-test** ***p*****-value**
AST (U/L)	178	32.1, 25.7–43.1	167	33.1, 27.5–40.7	0.604
		(2.0–689)		(10.5–178)	
ALT (U/L)	182	41.4, 30.0–73.4	167	41.5, 31.2–56.0	0.476
		(1.3–3026)		(6.9–765)	
ALP (U/L)	168	48.3, 32.3–84.4	167	42.0, 31.3–59.6	0.016
		(1.0–2015)		(15.0–1302)	
Total protein (g/L)	143	60, 56–64	166	59, 55–63	0.263
		(40–99)		(38–88)	
Glucose (mmol/L)^a^	152	4.6, 3.8–5.4	164	4.5, 3.6–5.3	0.320
		(1.1–19.3)		(1.3–17.2)	
Urea (mmol/L)^b^	184	5.4, 4.2–6.9	165	5.2, 4.3–6.5	0.663
		(1.2–44.6)		(2.0–39.0)	
Creatinine (μmol/L)^c^	184	88.4, 70.7–106.1	166	79.6, 70.7–97.2	0.023
		(26.5–539.2)		(44.2–592.3)	

a *To convert into mg/dL multiply by 18*.

b *To convert into mg/dL multiply by 6.03*.

c *To convert into mg/dL multiply by 0.011*.

In order to determine if observed changes were present only in patients with abnormal blood test results, we decided to select a subpopulation of dogs having parameters within normal values, which we believe mainly represents the undiagnosed dogs infected with skin dirofilariosis in real-clinical practice. Interestingly, in clinically healthy dogs, animals infected with *D. repens* had significantly reduced RBC (*p* = 0.025), Ht (*p* = 0.002), and lymphocyte (*p* = 0.031) count and significantly elevated glucose concentration (*p* = 0.023). ALP activity was boarder line elevated (*p* = 0.054) (Table 3).

**Table 3:** Hematological and biochemical parameters in clinically healthy dogs with blood check-up results within normal reference ranges.

**Parameter**	**Infected**		**Uninfected**		
	***n***	**mean ± SD (range)**	***n***	**mean ± SD (range)**	***t*-test*p*-value**
**Hematology**					
WBC (G/L)	93	11.89 ± 3.39 (5.1–18.4)	121	12.67 ± 3.65 (5.3–19.6)	0.110
Neutrophils (G/L)	93	8.33 ± 2.98 (1.6–16.2)	121	8.74 ± 3.23 (2.7–16.9)	0.343
Lymphocytes (G/L)	93	2.65 ± 1.46 (0.1–6.4)	121	3.07 ± 1.33 (0.6–7.0)	0.031
		2.3 (1.6–3.4)^a^		2.8 (2.1–4.0)^a^	0.007^b^
Eosinophils (G/L)	93	0.93 ± 0.87 (0–3.5)	121	0.86 ± 0.85 (0–5.6)	0.580
		0.7 (0.3–1.2)^a^		0.7 (0.3–1.1)^a^	0.645^b^
RBC (T/L)	93	7.03 ± 0.78 (5.2–8.9)	121	7.25 ± 0.69 (5.6–9.5)	0.025
Ht (%)	93	45.1 ± 4.5 (35–55)	121	47.0 ± 4.3 (36–56)	0.002
MCV (fl)	93	64.4 ± 3.8 (53–74)	121	65.1 ± 3.3 (58–79)	0.173
PLT (G/L)	93	320 ± 103 (110–598)	121	300 ± 98 (99–565)	0.136
**Biochemistry**					
AST (U/L)	84	31.9 ± 10.3 (13–66)	101	34.4± 10.1 (11-66)	0.095
ALT (U/L)	84	42.1 ± 19.2 (1–99)	101	42.4 ± 17.2 (14–94)	0.900
ALP (U/L)	82	54.3 ± 35.7 (1–163)	101	45.3 ± 23.5 (15–167)	0.054
		43 (30–63)^a^		40 (31–53)^a^	0.258^b^
Total Protein (g/L)	75	60.3 ± 5.8 (50–76)	100	60.2 ± 6.2 (50–77)	0.902
Glucose (mmol/L)^c^	80	4.76 ± 1.08 (1.6–7.6)	100	4.39 ± 1.07 (2.1–7.6)	0.023
Urea (mmol/L)^d^	85	5.29 ± 1.49 (3.0–12.0)	99	5.42 ± 1.46 (2.1–9.0)	0.537
Creatinine (μmol/L)^e^	85	83.83 ± 19.81 (23.9–130.0)	100	84.33 ± 20.84 (43.3–138.8)	0.868

a *Median (IQR)*.

b *Mann-Whitney U-test*.

c *To convert into mg/dL multiply by 18*.

d *To convert into mg/dL multiply by 6.03*.

e *To convert into mg/dL multiply by 0.011*.

## Discussion

*D. repens* is by far the most prevalent causative agent of human and canine dirofilariosis in Europe (1). It is generally believed that *D. repens* infections in dogs are asymptomatic, but several case reports (5–7) and the results of blood parameters in our study suggest that the illness is not non-pathogenic but goes rather undetected.

Underdiagnosis of skin dirofilariosis is mainly due to the lack of diagnostic tools to detect occult infections. Amicrofilaremic infections can occur after previous antiparasitic treatment based on macrocyclic lactones, long prepatent time of the infection or monosex infection. Microfilariae periodicity and low intensity of microfilaremia also contribute to obtaining false negative results while investigating a dog for skin dirofilariosis. In contrast, clinicians are left to their own devices for diagnosing and interpreting importance of *D. repens* infections. Thus, having hematological and biochemical biomarker to suggest the presence and severity of infection might be a supportive tool for managing the disease progression, especially in asymptomatic patients.

Our first analysis of all dogs admitted to the study mirrored the real-clinical scenario of dogs brought for different reasons and showed that dogs additionally infected with *D. repens* compared to those uninfected had significantly lower RBC, thrombocyte, and lymphocyte counts, decreased Ht, and increased ALP activity.

The second analysis included a subpopulation of dogs having parameters within normal reference ranges, and in this group, differences were observed in infected animals, such as lower RBC, Ht, lymphocytes, and higher glucose and elevated boarder line ALP activity comparing to the uninfected group. Despite presenting as clinically healthy dogs, these alterations indicate that *D. repens* infection influenced the functioning of their body.

Statistically significant hematological alterations and ALP increase in dogs infected with *D. repens* are similar to those observed in other filarial disease, especially *D. immitis* infections (13), so the pathogenicity, as claimed by other authors (5, 14), is rather correlated with Mf than the presence of adult parasites.

The lower RBC, hematocrit, and hemoglobin characterize anemia in dogs and may be a result of destructive capability of Mf associated with severe intravascular hemolysis as reported in dogs infected with *Diptelonema reconditum* (15). It also might be connected to inflammation-associated production of pro-inflammatory cytokines resulting in suppression of erythrocytes production and inhibition of iron absorption and utilization (16). Lower thrombocyte count may be due to platelet consumption or presence of antiplatelet antibodies observed in dogs infected with other vector-borne diseases (17) or might be assigned to immune-mediated platelet destruction as reported in dogs infected with *D. immitis* (13). Lymphopenia was not only observed in infected patients in this study but also reported in another dog infected with *D. repens* (5) and during other canine-borne vector diseases. The mechanism of lymphopenia might be associated to endogenous glucocorticoids release that occurs in response to infectious stimuli or stress, which leads to lymphocyte apoptosis or sequestration of lymphocytes in lymphoid organs (18).

The increase in creatinine was observed in the first analysis in the infected group compared to the uninfected group in our study but was not shown to be statistically significant in dogs infected with *D. immitis*. On the contrary, another parameter suggesting renal damage (urea) was increased in *D. immitis*-infected patients but not in our *D. repens*-infected group (13). A case report describing the most severe course of skin dirofilariosis of a dog coinfected with *D. immitis* suggested that only *D. repens* Mf were involved in kidney damage. However, other reports suggest that microfilaremic (not occult) dogs infected with *D. immitis* show marked signs of kidney damage (14, 19, 20).

ALP was significantly higher in infected dogs. Although not being highly liver specific (21), ALP is a marker having a good sensitivity for liver diseases in dogs, suggesting cholestasic disease or chronic hepatitis/cirrhosis. Other parameters suggesting liver injuries, as ALT and AST were reported to be increased in case reports of dogs infected with *D. repens* (5, 22) and in some dogs in our study. This difference, however, was not statistically significant while comparing the infected and uninfected group, suggesting a rather big variability between infected individuals. Interestingly, the ALP increase was also the only liver parameter changed in *D. immitis-*infected dogs (13). The increase in ALP activity, with AST and ALT activity within normal values, makes a hepatocellular damage unlikely and might be rather due to chronic stress associated with increase in endogenous glucocorticoids (23), although, in patients having elevated AST, ALT, and ALP activity, an injury of the liver was very likely.

A higher level of glucose was observed in dogs infected with *D. repens* having all blood parameters within normal ranges. This also may be the result of higher glucocorticoids levels in these animals, which again suggests a correlation between *D. repens* and development of chronic stress response. Interestingly, glucose level was not significantly changed while the whole population was analyzed, which might suggest that, after development of concomitant disease, the host's body reaction to the infection changes.

Sometimes parasitological coinfection might positively influence the course of the main disease. For example, it has been reported that coinfection of malaria and hookworms seems to increase malaria incidence but at the same time might protect from malaria severe manifestations in humans (24). Moreover, dogs infected solely with *Babesia canis* compared to those coinfected with *D. repens* displayed more pronounced biochemical changes, implying coinfection with *D. repens* was somehow beneficially counteracting against renal and liver damage characteristic for the course of babesiosis in dogs (9), although in the same study, the authors reported that thrombocytopenia and anemia concomitant with babesiosis in dogs were aggravated in individuals coinfected with *D. repens*. Our results show differences in blood parameters between seemingly asymptomatic *D. repens* patients and uninfected dogs. *D. repens* infection seems to promote glucocorticoids release as chronic stress response associated with lymphopenia, glucose level, and ALP activity increase that may predispose infected individuals to develop other infectious, metabolic, or hormonal diseases in the future. This in turn might explain why dogs presented with different diseases and at the same time coinfected with *D. repens* had more serious alternations in blood parameters than uninfected dogs. We suspect that the chronic stress response observed in seemingly healthy dogs may lead to more deepened alternations of concomitant diseases in the future.

There are several limitations in the presented study, such as no checkups or no final diagnosis of concomitant disease available for all admitted dogs. However, we aimed to show a trend in blood parameter changes related to *D. repens* infection. In dogs with different ailments as well as in asymptomatic dogs, significant differences in blood parameters were noted. Our data suggest that *D. repens* infection indeed induces some biological changes in the host. The length of the infection is not known either, but in naturally infected dogs, it cannot be evaluated. However, this mirrors more adequately the real clinical approach to the problem of increasing spread of *D. repens* parasite.

Our results indicate that *D. repens* infection complicates concomitant diseases, and animals develop more serious and detectable changes such as lower numbers of erythrocytes, lymphocytes, and thrombocytes and higher activity of ALP and creatinine. Even among dogs with blood results within normal reference ranges, individuals infected with *D. repens* showed detectable differences compared to uninfected ones, suggesting that seemingly healthy animals may still have altered biological processes or early indications of disease. The analysis of parameters within dogs having results within normal reference ranges, where the differences between groups are indeed within normal values, suggests a trend and perhaps a predisposition of development of other diseases in the future. Further studies with cortisol level evaluation, the measurements of total but also differentiation between liver ALP (L-ALP) and corticosteroid ALP (C-ALP) isoforms in infected dogs with full follow-ups could be valuable.

## Conclusions

Our paper describes hematological and biochemical changes in dogs naturally infected with *D. repens*. Alterations such as a decrease in RBC, lymphocytes, thrombocytes, hematocrit, as well as increases in ALP and creatinine could be indicators of coinfection with *D. repens*, especially in endemic regions. Furthermore, the results in clinically healthy dogs suggest that the presence of *D. repens* might lead to development of anemia and a state of chronic stress response, characterized by the combination of lymphopenia and increase in glucose level and ALP activity and, as such, predispose infected dogs to other ailments. This could explain the existence of more serious statistically significant blood parameters alternations in dogs with different ailments simultaneously coinfected with *D. repens*.

Our results strongly indicate that *D. repens* infection has a pejorative influence on the health of the canine host independent of their clinical condition. Finally, the pathogenicity of *D. repens* remains a mystery but needs further investigation in order to comprehend its association with chronic stress response in infected individuals.

## Data Availability Statement

The raw data supporting the conclusions of this article will be made available by the authors, without undue reservation.

## Ethics Statement

Ethical review and approval was not required for the animal study because the blood samples were collected for direct benefit of the patients and the analysis was performed on leftover samples (Act of 15th January 2015 on the protection of animals used for scientific purposes). The director of veterinary services of local dog shelters and the owners of privately owned dogs were informed about the results of Dirofilaria testing of the dogs under their care. Written informed consent for participation was not obtained from the owners because no interventions outside routine care were performed. All blood samples were taken during routine checkups or process of diagnosis in veterinary clinics and only then the results were used to compare the results between *D. repens* infected and uninfected groups of dogs. Multiplex PCR was run on the leftover blood samples. A verbal consent of the owners' to voluntirely test their animals for skin dirofilariosis was present for all dogs admitted in the study. All data were de-indentified before running statistical analysis and the entire anonymity of data has been assured. The director of local dog shelters and the owners of client-owned dogs were informed about the results of Dirofilaria testing of the dogs under their care. Doctors of veterinary medicine were provided with all information that could help them to introduce the best treatment to infected dogs.

## Author Contributions

AD and MK collected clinical data, parasitic, biological specimens, and performed blood smears. ED and MEW performed PCR. MC performed the statistical analysis of the results. MEW, AD, MK, ED, PJ, and MW planned and discussed the study and the results. MEW wrote the manuscript. All authors read and approved the final version of the manuscript.

## Conflict of Interest

The authors declare that the research was conducted in the absence of any commercial or financial relationships that could be construed as a potential conflict of interest.

## Abbreviations

ALP: alkaline phosphatase
ALT: alanine aminotransferase
AST: aspartate transaminase
EDTA: ethylene diamine tetraacetic acid
MCH: mean corpuscular hemoglobin
MCHC: mean corpuscular hemoglobin concentration
MCV: mean corpuscular volume
PCR: polymerase chain reaction
RBC: red blood cell
PMN: polymorphonuclear leukocytes
WBC: white blood cells: leukocytes
Mf: microfilariae.
